# The Healing of Arteries after Ligature in Man and Animals

**Published:** 1887-03

**Authors:** 


**The Healing of Arteries after Ligature in Man and Animals.** By J. Collins Warren, M. D., Assistant Professor of Surgery, Harvard University; Surgeon to the Massachusetts General Hospital; Member American Surgical Association; Honorary Fellow Philadelphia Academy of Surgery. One volume. 184 pages. Parchment muslin binding. Price, $3.25. New York: William Wood & Co.

After careful study, investigation and examination of many specimens, the writer has compiled this complete book. It is, so far as we know, the only complete monograph on the subject. "Hitherto the study has been carried on in a more or less fragmentary way, different portions of it having received very minute attention and from the hands of the ablest pathologists. The attempt has been made here to study the question from a more comprehensive standpoint, to observe not only the behavior of the various tissues concerned in the process of repair, but also the different phases through which the vessel passes from the moment of the ligature until the condition is reached, after which no further change occurs." The book is illustrated with twelve full-page plates in black and colors.

